# A20 regulates canonical wnt-signaling through an interaction with RIPK4

**DOI:** 10.1371/journal.pone.0195893

**Published:** 2018-05-02

**Authors:** Brooke N. Nakamura, Alison Glazier, Michael G. Kattah, Bao Duong, Yanxia Jia, Daniel Campo, Ling Shao

**Affiliations:** 1 Department of Medicine, Division of Gastroenterology and Liver Diseases, University of Southern California Keck School of Medicine, Los Angeles, California, United States of America; 2 Department of Medicine, Division of Gastroenterology, University of California, San Francisco, California, United States of America; 3 Department of Biological Sciences, University of Southern California, Los Angeles, California, United States of America; Johns Hopkins School of Medicine, UNITED STATES

## Abstract

A20 is a ubiquitin-editing enzyme that is known to regulate inflammatory signaling and cell death. However, A20 mutations are also frequently found in multiple malignancies suggesting a potential role as a tumor suppressor as well. We recently described a novel role for A20 in regulating the wnt-beta-catenin signaling pathway and suppressing colonic tumor development in mice. The underlying mechanisms for this phenomenon are unclear. To study this, we first generated A20 knockout cell lines by genome-editing techniques. Using these cells, we show that loss of A20 causes dysregulation of wnt-dependent gene expression by RNAseq. Mechanistically, A20 interacts with a proximal signaling component of the wnt-signaling pathway, receptor interacting protein kinase 4 (RIPK4), and regulation of wnt-signaling by A20 occurs through RIPK4. Finally, similar to the mechanism by which A20 regulates other members of the receptor interacting protein kinase family, A20 modifies ubiquitin chains on RIPK4 suggesting a possible molecular mechanism for A20’s control over the wnt-signaling pathway.

## Introduction

Tumor necrosis factor alpha induced protein 3 (TNFAIP3), also known as A20, is a ubiquitin editing enzyme with well-known functions regulating inflammatory signaling and cell death downstream of the TNF-receptor superfamily [1]. Absence of this critical negative regulatory protein recapitulates the phenotype of many inflammatory diseases in mice [2] and loss of function mutations leads to severe auto-inflammatory disease in humans [3]. Moreover, somatic mutations in A20 have been found in several B-cell malignancies suggesting a potential role as a tumor suppressor as well [4].

The known functions of A20 rely upon binding and modifying ubiquitin chains on target proteins. For example, A20 can remove K63-linked ubiquitin moieties through an ovarian tumor-like domain, while the fourth zinc finger has ubiquitin E3-ligase activity and can add K48-linked ubiquitin chains to proteins promoting their proteasome-dependent degradation [5,6]. More recently, the seventh zinc finger of A20 has been shown to specifically bind linear ubiquitin chains present in the proximal TNF-receptor signaling complex which is important for the interaction with its targets [7–9].

Molecular targets of A20 include proximal signaling elements such as TRAF1/2 downstream of TNF and CD40 [10], and TRAF6 downstream of IL-1 [11,12]. One well-known target of A20 function is the receptor interacting protein kinase (RIPK) family [5,13,14]. This family of proteins consists of seven members in humans. A20 regulates TNF/IL-1/TLR signaling through RIPK1 and NLR signaling through RIPK2 [12,15–17]. More recently, A20 has been shown to regulate pyroptotic and necroptotic signaling through an interaction with RIPK3 [18,19].

Receptor interacting protein kinase 4 (RIPK4) is the fourth member of the RIPK family which shares significant homology with the other RIPK family members, particularly within the kinase domain [20]. Mutations in RIPK4 are responsible for a rare autosomal-recessive disease called popliteal pterygium syndrome, also known as Bartsocas-Papas syndrome [21,22]. RIPK4 global knockout mice show a defect in gastric tube development [23]. In this context, RIPK4 has been shown to positively regulate beta-catenin activity downstream of wnt-signaling. RIPK4 knockout mice also have a thickened epidermis due to abnormal keratinocyte differentiation [24], while keratinocyte-cell specific RIPK4 knockout impairs epithelial barrier function [25].

We previously showed that mice with an intestinal-epithelial cell specific deletion of A20 on an APCmin background developed larger and more numerous colonic tumors [26]. Indeed, A20 expression in human colorectal adenomas and carcinomas has been reported to be decreased [27,28]. Surprisingly, we found that A20 might have a direct role on regulating the wnt-signaling pathway. Although we showed that A20 interacts with axin, a central scaffolding molecule in the wnt-signaling cascade and supported beta-catenin ubiquitination, a precise molecular mechanism for this regulation remains unknown. Given the prior known associations between A20 and the RIPK family of proteins, we hypothesized that the regulation of wnt-signaling by A20 therefore might proceed through RIPK4. In this report, we show for the first time that A20 interacts with RIPK4 and that the regulation of wnt-signaling by A20 is dependent on RIPK4. Mechanistically, A20 appears to support K48-linked ubiquitination of RIPK4 after wnt-stimulation, analogous to its role in regulating RIPK1 in the context of TNF signaling.

## Methods

### Cell lines, antibodies and reagents

RKO (CRL-2577) and 293 (CRL-1573) cells were obtained from the ATCC, grown at 37°C at 5% CO2 and routinely maintained in DMEM (Genesee Scientific, San Diego, CA) containing 10% FCS (Seradigm/VWR, Radnor, PA) and 1% Penicillin/Streptomycin/L-glutamine (Caisson, Smithfield, UT). Recombinant wnt3a, wnt5a, and TNF-α (TNF) were obtained from R&D Systems (Minneapolis, MN). For stimulation, unless otherwise noted, TNF was used at a concentration of 10ng/mL, wnt3a was used at a concentration of 50ng/mL and wnt5a was used at a concentration of 100ng/mL. Antibodies to A20 (Clones B5 and 4H16) and beta-catenin (Clone C18) were obtained from Santa Cruz Biotechnology (Santa Cruz, CA). Antibodies to RIPK4 and Dishevelled-2 were obtained from Cell Signaling (Danvers, MA). Antibodies to FLAG were obtained from Sigma (St. Louis, MO). Human cDNA clones (Dharmacon, Lafayette, CO) were used to clone full length and truncation mutants of A20, RIPK4, and K48-only ubiquitin into PCMV3Tag Vectors (Agilent, Santa Clara, CA). An A20 zinc-finger 4 mutant plasmid (C624A/C627A) previously shown to be defective in forming polyubiquitin chains [29] was generated by site directed mutagenesis using the QuikChange Mutagenesis Kit (Agilent, Santa Clara, CA). RIPK4 siRNA was obtained from Bioneer (Alameda, CA). Transfections were performed with Lipofectamine 2000 (Invitrogen, Carlsbad, CA) according to the manufacturer’s instructions.

### Generation of knockout cell lines

**Fig 1:**
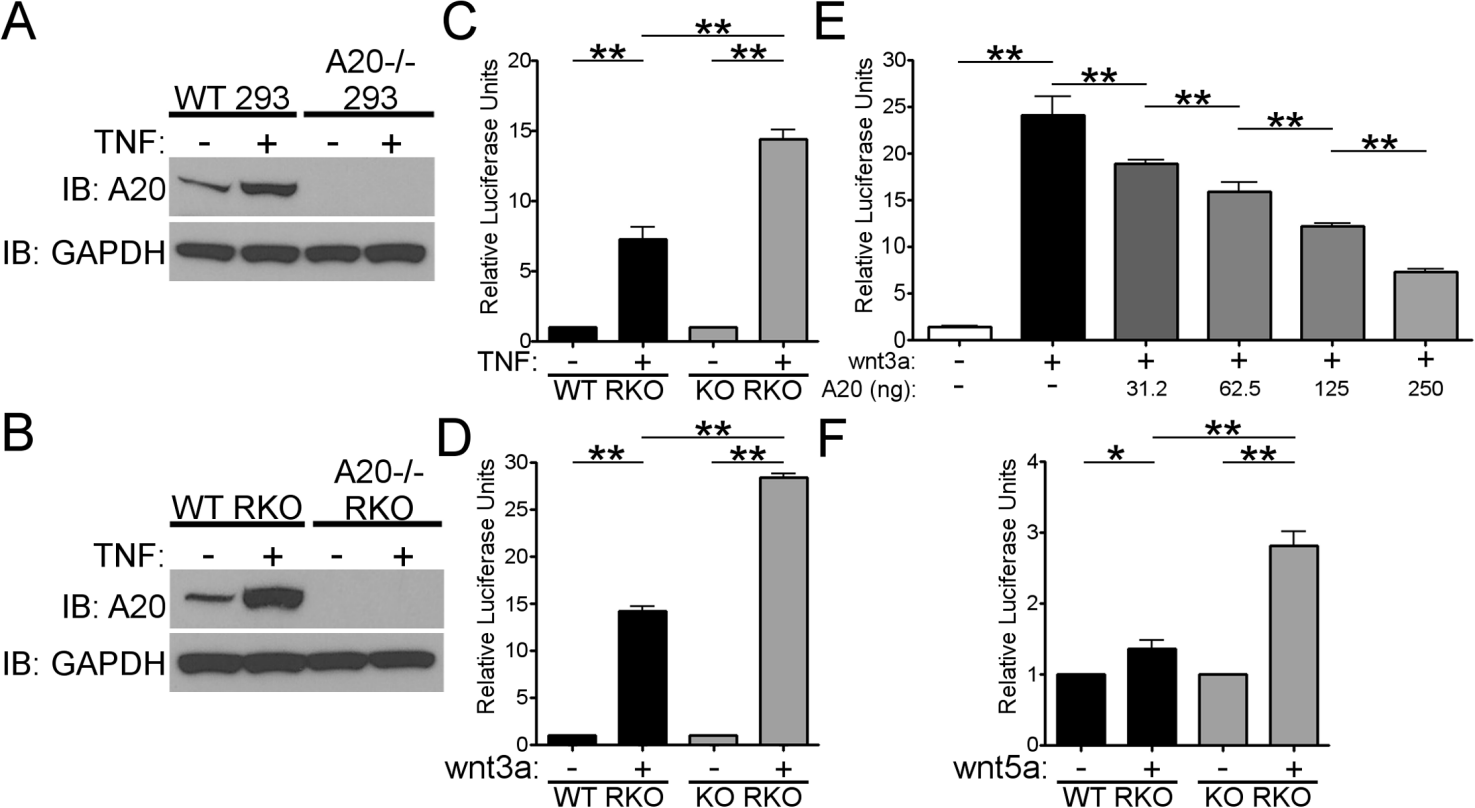
Dysregulation of TNF and wnt-signaling in A20 knockout cell lines. A) Wild-type (WT 293) and A20 knockout 293 (A20-/- 293) cells were stimulated with TNF for 4 hours. Lysates were then blotted for A20 or GAPDH as a loading control. B) Wild-type (WT) RKO and A20 knockout (A20-/-) RKO cells were stimulated with TNF for 4 hours. Lysates were then blotted for A20 or GAPDH as a loading control. C) Wild-type (WT RKO) or knockout RKO (KO RKO) were transfected with an NFkB-luciferase reporter and then stimulated with TNF for 8 hours. Luciferase activity was measured and normalized to Renilla luciferase. D) Wild-type (WT RKO) or knockout RKO (KO RKO) were transfected with a TCF4-luciferase reporter and then stimulated with wnt3a for 8 hours. Luciferase activity was measured and normalized to Renilla luciferase. E) A20-/- 293 cells were transfected with a plasmid expressing wild-type A20 in increasing concentrations along with a TCF4-luciferase reporter. Cells were stimulated with wnt3a for 8 hours and luciferase-reporter activity measured and normalized to Renilla luciferase. F) WT RKO or A20-/- RKO cells were transfected with a TCF4-luciferase reporter and then stimulated with wnt5a for 8 hours. Luciferase activity was measured and normalized to Renilla luciferase. * = p < 0.05, ** = p < 0.01. Each panel is representative of at least three independent experiments.

A20 knockout cell lines were generated using transcription activator-like effector nucleases (TALEN) according to published protocols [30]. Monomer and TALEN plasmids were obtained from Addgene (Cambridge, MA). The targeting sequences used were 5’-TGCCTCATGCATGCCACTTC-3’ and 5’- TTCAGGACACAGACTTGGTA-3’. Cells were transfected with 2ug of each TALEN plasmid with a puromycin selection cassette. Transfectants were first selected using 2ug/mL puromycin for 3 days and then limiting dilution performed. Knockout clones were then screened by qPCR and Western blot (Fig 1 and S1 Fig).

### Quantitative PCR

Total RNA was isolated from cells using Trizol reagent (Invitrogen, San Diego, CA) according to the manufacturer’s instructions. Reactions were run using the One-Step SYBR Primescript RT-PCR Kit II (Clontech, Mountain View, CA) on an ABI 7900HT (Applied Biosystems). All qPCR reactions were run in triplicate and normalized to the expression of housekeeping genes and analyzed using the ddCT method. The primers used for qPCR were: A20 5’-TCATGCATGCCACTTCTCAG-3’ and 5’-TGCGTGTGTCTGTTTCCTTG-3’ and HPRT1 5’- CCTGGCGTCGTGATTAGTGAT-3’ and 5’-AGACGTTCAGTCCTGTCCATAA-3’.

### Mammalian yeast-2-hybrid

Human A20 and RIPK4 or controls were cloned upstream of the GAL4 DNA-binding domain and the VP16 activation domain containing plasmids using the Matchmaker Mammalian Two-Hybrid System (Clontech, Mountain View, CA). These plasmids in addition to the reporter plasmid containing a GAL4 –responsive element upstream of a secreted alkaline phosphatase reporter gene were transfected to RKO cells. Secreted alkaline phosphatase activity was assayed using a SEAP Chemiluminescence detection kit (Clontech) on a Fluostar Omega microplate reader (BMG Labtech, Cary, NC).

### Luciferase reporter assay

The MegaTOPFlash TCF4-luciferase reporter plasmid was a kind gift from R. Nusse (Stanford University, CA). Luciferase assays were performed as previously described [31]. Briefly, 500ng of reporter plasmids was co-transfected with control plasmid, RIPK4 K51R plasmid, control siRNA or RIPK4 siRNA (Thermo Fisher Scientific, Waltham, MA). After appropriate stimulation, cells were lysed in 1% Triton-X100 buffer and developed.

### Western blot

Cells were lysed in 1% Triton X-100 containing protease inhibitors (Complete EDTA-free, Thermo Fisher Scientific, Waltham, MA), 150mM NaCl, and 10% Glycerol for 30 minutes at 4 degrees. Lysates were cleared by centrifugation and protein concentrations determined by BCA Assay (Thermo Fisher Scientific, Waltham, MA). For immunoprecipitation, 1milligram of total protein was incubated with 3 micrograms of antibody overnight at 4 degrees. Protein G agarose beads were added and incubated for 1 hour at 4 degrees then washed 5x in lysis buffer. Beads were boiled in SDS-PAGE loading buffer (Invitrogen) and then resolved on 4–12% Bis-Tris SDS-PAGE gels.

### Library prep and sequencing

Total RNA was isolated using RNeasy Plus Mini Kit (Qiagen, Hilden, Germany) and was checked for degradation in a Bioanalyzer 2100 (Agilent, Santa Clara, CA, USA). RNA quality was very high for all samples, with RIN numbers varying between 7.9 and 10. RNAseq libraries were prepared from 500 ng of total RNA a using a Kapa mRNA HyperPrep Kit for Illumina platforms (Kapa Biosystems, Inc., Wilmington, MA, USA) which isolates mRNA via poly(A) capture. Final library products were quantified using the Qubit 2.0 Fluorometer (Thermo Fisher Scientific Inc., Waltham, MA, USA), and the fragment size distribution was determined with the Bioanalyzer 2100. The libraries were then pooled equimolarly, and the final pool was quantified via qPCR using the Kapa Biosystems Library Quantification Kit, according to manufacturer’s instructions. The pool was sequenced in an Illumina HiSeq 2500 platform (Illumina, San Diego, CA, USA), in Rapid Single-Read 75 cycles format, targeting at least 30 million reads per sample. The preparation of the libraries and the sequencing was performed at the UPC Genome Core (University of Southern California, Los Angeles, CA, USA). Raw data files are available from the Genome Expression Omnibus (GEO Database), accession number: GSE111084.

### Bioinformatics analysis

Initial read quality and adaptor content of FASTQ files were assessed with FastQC [32]. Reads were then trimmed based on quality score, and adaptor sequences removed using Trimmomatic [33]. After filtering, surviving reads were checked again in FastQC to ensure that only high-quality transcriptome reads were put into the analysis pipeline. These high-quality reads were mapped to the human genome (ver. GRCh38.p7) using the ultra-fast aligner STAR [34]; the same software was used to obtain uniquely mapping read counts for each gene feature included in a Gene Transfer Format (GTF) file. Both the genome and the GTF file were downloaded from the GENCODE database (https://www.gencodegenes.org).

Differential gene expression analysis was performed with the R/Bioconductor package DESeq2, using as input the raw counts obtained in the previous step. Other statistical analysis and plots were also done in R ver. 3.3.3 and RStudio ver. 1.0.136 [35].

### Statistical analysis

Statistical analysis was performed with Graphpad Prism 4 (Graphpad Software, San Diego, CA). Comparisons between two groups were performed by two-tailed unpaired Student’s t-test. Multigroup comparisons were performed by one-way analysis of variance (ANOVA). p< 0.05 was used as the threshold for statistical significance. All experiments shown are representative of at least three independent experiments.

## Results

### A20 knockout cell lines show exaggerated canonical wnt-signaling

We previously demonstrated that A20 siRNA knockdown enhanced canonical wnt-signaling in a colon cancer cell line, RKO, with intact beta-catenin signaling [26]. To further study the role of A20 in the wnt-signaling pathway we generated RKO and 293 cells deficient for A20 using genome editing technology. We confirmed the knockout of A20 by western blot (Fig 1A and 1B) and qPCR (S1A and S1B Fig). As expected, consistent with the role of A20 in regulating TNF-induced NFkB, A20 knockout RKO and 293 cells showed exaggerated NFkB luciferase reporter activity after stimulation with TNF (Fig 1C and S1C Fig). Furthermore, congruent with our previous data, both knockout cell lines also showed increased wnt3a dependent TCF4-reporter luciferase activity (Fig 1D and S1D Fig). Reconstitution of A20 in knockout 293 cells suppressed wnt3a-dependent TCF4-luciferase reporter activity in a dose-dependent fashion (Fig 1E and S1E Fig). Additionally, A20 knockout RKO cells showed an increase in TCF4-luciferase reporter activity in response to the non-canonical wnt5a (Fig 1F). In concert with our prior results using an siRNA approach, our novel A20 knockout cell lines demonstrated enhanced canonical wnt-signaling compared to wild-type cells. As expected, they also demonstrate increased TNF-dependent NFkB activity consistent with the known role of A20 in restricting TNF-receptor signaling.

### Wnt-dependent gene expression is perturbed in the absence of A20

**Fig 2:**
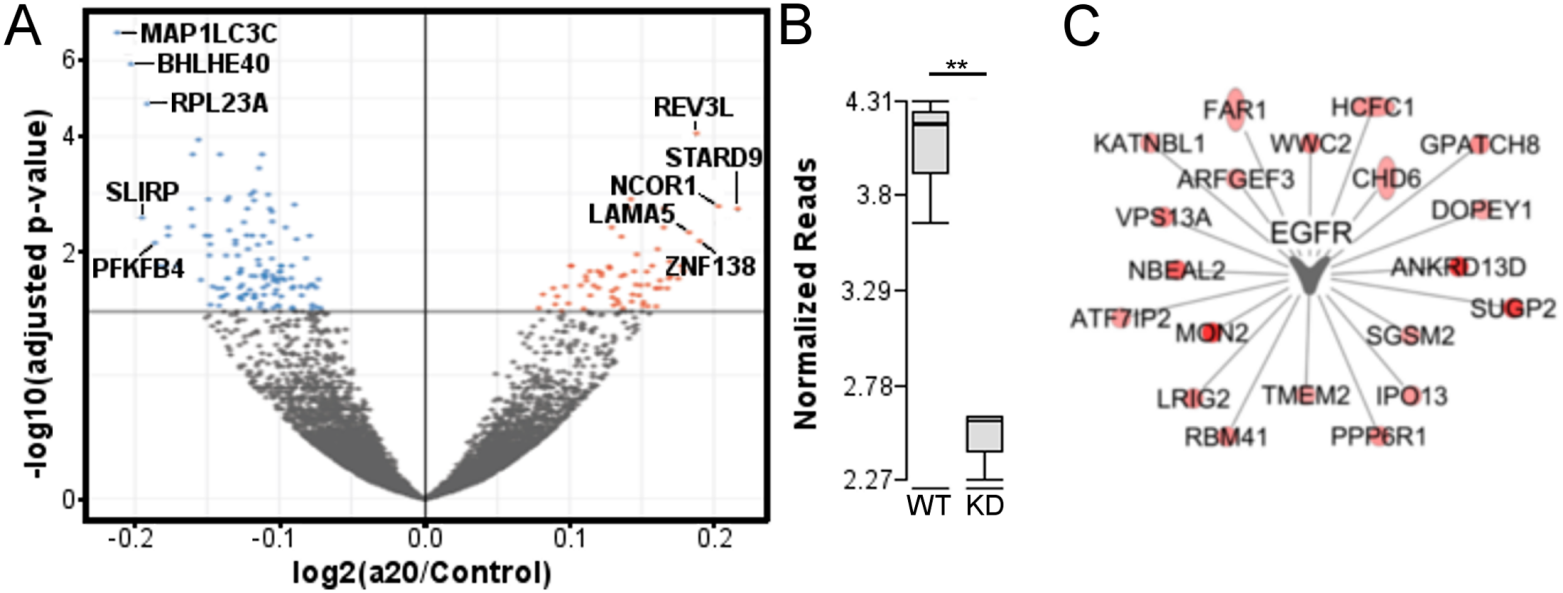
Gene expression changes after wnt3a stimulation in the presence or absence of A20. A) Volcano plot showing the differentially downregulated (blue) and upregulated (red) genes in A20 knockdown versus control knockdown RKO after 24 hours of treatment with wnt3a. The top five upregulated and downregulated genes are labelled. B) RNA expression of A20 in control (WT) or A20 siRNA knockdown (KD) C) Pathway analysis showing dysregulation of genes related to EGFR signaling in RKO cells stimulated with wnt3a after A20 siRNA knockdown.

Since A20 seemed to influence wnt3a-dependent transcriptional activity, we investigated specific genes regulated by A20 after wnt3a stimulation. Using RKO cells with intact beta-catenin signaling we knocked down A20 using a specific siRNA and then stimulated the cells with wnt3a for 24 hours and then performed RNAseq. A volcano plot of differentially expressed genes between control and A20 siRNA treated cells highlights the top differentially upregulated and downregulated genes (Fig 2A). A list of the top ten upregulated and downregulated genes is presented in S2 Fig. As expected, A20 transcripts were significantly downregulated by treatment with an A20 siRNA compared with control siRNA (Fig 2B). Interestingly, pathway analysis of differentially regulated genes showed that many of the transcripts dysregulated in the absence of A20 revolved around signaling components downstream of epidermal growth factor receptor (EGFR) (Fig 2C). These data suggest that A20 negatively regulates multiple transcripts downstream of wnt3a, ultimately leading to potential alterations in EGFR signaling. Further studies are needed to determine the role of A20 in regulating the EGFR pathway.

### A20 interacts with RIPK4

**Fig 3:**
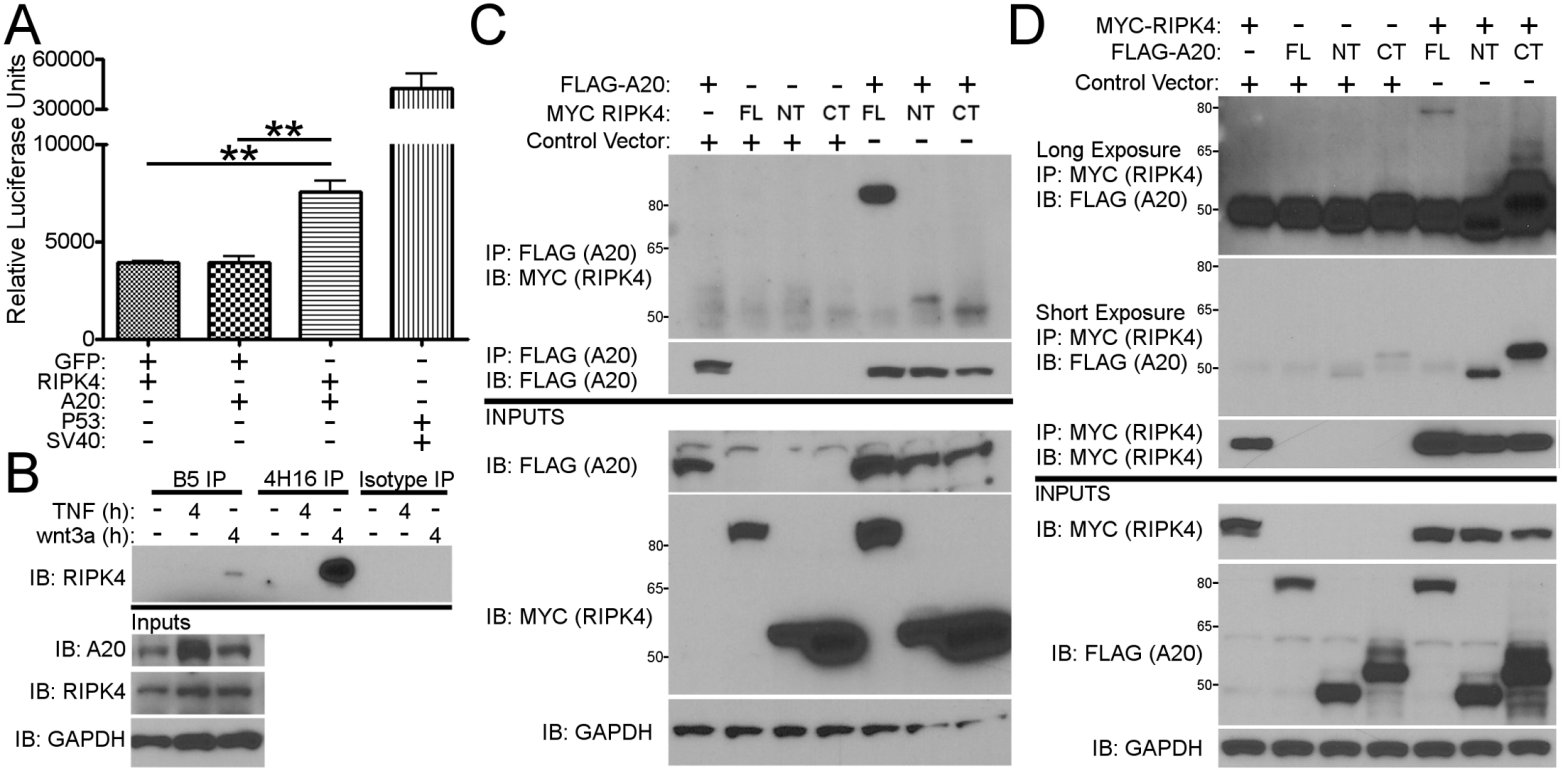
A20 interacts with RIPK4. A) Mammalian-2-hybrid assay showing interaction of A20 and RIPK4, but not A20 with GFP (as a negative control) or RIPK4 with GFP (as a negative control). Interaction of p53 and SV40 shown as a positive control. B) Wild-type RKO cells were stimulated with either TNF or wnt3a for 4 hours. Cells were lysed and subject to immunoprecipitation of A20 using two separate antibodies (B5 or 4H16) or an isotype control. Immunoprecipitates were subject to Western blot for RIPK4. Inputs for A20, RIPK4, and GAPDH as a loading control are shown below. C) Flag-tagged A20 (FLAG-A20), MYC-tagged full-length RIPK4 (FL), RIPK4 N-terminal domain (NT), RIPK4 C-terminal domain (CT), and control vector were expressed in RKO cells. Cell lysates were subject to immunoprecipitation with an antibody towards MYC and then blotted with an antibody against FLAG (upper) or immunoprecipitation with an antibody towards FLAG and then blotted with an antibody against MYC (lower). Inputs shown below. GAPDH shown as a loading control. D) MYC-tagged RIPK4, FLAG-tagged full-length A20 (FL), FLAG-tagged A20 N-terminal domain (NT), FLAG-tagged A20 C-terminal domain (CT), and a control vector were expressed in RKO cells. Cell lysates were subject to immunoprecipitation with an antibody towards MYC and then blotted with an antibody against FLAG. Inputs shown below. GAPDH shown as a loading control. ** = p < 0.01. Each panel is representative of at least three independent experiments.

RIPK4 shares significant homology with other members of the RIPK family raising the possibility that A20 might interact and regulate RIPK4 in a similar manner that it does with RIPK1, -2, and -3 (S3 Fig). We first used a mammalian yeast-2-hybrid system to explore whether A20 and RIPK4 might interact *in vitro*. Overexpression of A20 fused to the GAL4 DNA-binding domain showed significant activation of a secreted alkaline phosphatase reporter when co-transfected with RIPK4 fused to the VP16-transactivation domain (Fig 3A). No activity of the reporter was found when A20 was co-expressed with GFP-fused to the VP16-transactivation domain. Similarly, no activation of the reporter was observed when GFP-fused to the GAL4 DNA-binding domain was co-expressed with RIPK4 fused to the VP16-transactivation domain. As a positive control, SV40 fused to the GAL4 DNA-binding domain showed significant activation of the secreted alkaline phosphatase reporter when co-expressed with p53 fused to the VP16-transactivation domain.

We reasoned that the constitutive interaction between A20 and RIPK4 observed in the mammalian-2-hybrid system might be due to the non-physiologic levels achieved by overexpression. We therefore sought to confirm this interaction between endogenous proteins. First, we noted that while stimulation of RKO cells with TNF lead to a significance increase in A20 protein after two hours, TNF stimulation did not increase levels of RIPK4 up to 8 hours after stimulation. Furthermore, stimulation with wnt3a did not increase levels of A20 or RIPK4 protein up to 8 hours after stimulation (S4A Fig). To investigate a potential physiologic interaction, we immunoprecipitated A20 in RKO cells and performed a western blot for RIPK4. Two A20-specific antibodies, but not an isotype control antibody, were able to co-immunoprecipitate RIPK4 with endogenous A20 after stimulation with recombinant human wnt3a, but not TNF (Fig 3B). These data suggest that similar to the interaction between A20 and other RIP kinase family members, A20 interacts with RIPK4 only after specific stimuli, in this case wnt3a.

To further define the specificity of the interaction, we generated N-terminal and C-terminal truncation mutants of RIPK4 and A20 to narrow down the domains of interaction between the two proteins (S5 Fig). Full-length A20 only efficiently interacted with full-length RIPK4, suggesting perhaps a conformational dependent interaction motif present on RIPK4 (Fig 3C). In contrast, full-length RIPK4 interacted with both N-terminal and C-terminal truncation mutants of A20, suggesting multiple domains of interaction (Fig 3D). Indeed, while the interaction between A20 and RIPK1 and RIPK2 are thought to occur in the intermediate domain the interaction motifs are not well defined.

### A20 regulates wnt-signaling through RIPK4

**Fig 4:**
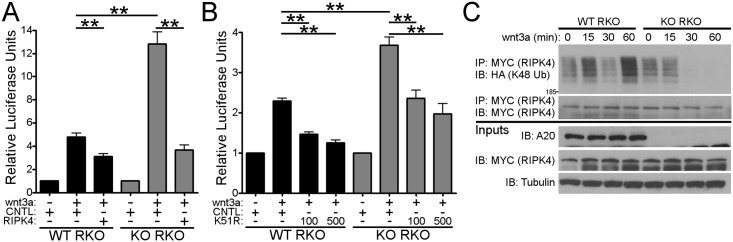
Regulation of wnt-signaling by A20 through RIPK4. A) Wild-type (WT RKO) or A20 knockout RKO (KO RKO) were transfected with a TCF4-luciferase reporter and either a control or RIPK4 siRNA. Cells were stimulated with wnt3a for 8 hours. Luciferase activity was measured and normalized to Renilla luciferase. B) Wild-type (WT RKO) or A20 knockout RKO (KO RKO) were transfected with a TCF4-luciferase reporter and either a control or a RIPK4-K51R plasmid at different concentrations. Cells were stimulated with wnt3a for 8 hours. Luciferase activity was measured and normalized to Renilla luciferase. C) Wild-type (WT RKO) or A20 knockout RKO (KO RKO) were transfected with MYC-tagged RIPK4 and an HA-tagged K48-only ubiquitin construct. Cells were stimulated with wnt3a for the indicated time points. Lysates were subjected to immunoprecipitation with an antibody against MYC and then blotted with an antibody against HA. Inputs are shown below. Tubulin is shown as a loading control. ** = p < 0.01. Each panel is representative of at least three independent experiments.

To determine whether dysregulation of wnt-signaling in A20 deficient cells was dependent on RIPK4, we performed the same analysis in the presence of a RIPK4 siRNA or a control siRNA. To directly determine whether the enhanced canonical and non-canonical signaling in the absence of A20 was due to dysregulation of RIPK4, we used a RIPK4 specific siRNA to knockdown RIPK4 protein expression and then stimulated cells with recombinant human wnt3a and assayed for luciferase reporter activity. Indeed, siRNA knockdown of RIPK4 in A20 knockout cells partially reversed the enhanced wnt3a-dependent TCF4 luciferase reporter activity (Fig 4A and S4B Fig).

RIPK4 is known to positively regulate the canonical wnt-signaling pathway by phosphorylating two residues on Dishevelled-2 [23]. A kinase dead mutant of RIPK4 (RIPK4 K51R) is not able to phosphorylate Dishevelled-2 and acts as a dominant negative regulator of canonical wnt-signaling. We therefore tested whether overexpression of the RIPK4 K51R dominant negative construct could similarly inhibit the enhanced canonical wnt-signaling seen in A20 deficient cells. Congruent with these results, overexpression of a dominant negative kinase dead mutant of RIPK4 (K51R) in A20 knockout RKO cells also partially abrogated the enhanced wnt3a-dependent TCF4 reporter activity in a dose-dependent manner (Fig 4B and S4C Fig). Taken together these data suggest that A20 normally restricts the both the canonical and non-canonical wnt-signaling pathway, in part, through an interaction with RIPK4.

Finally, A20 is well-known to regulate the RIPK family by modifying their associated ubiquitin chains. To investigate whether A20 might have similar activity on RIPK4 we overexpressed MYC-tagged RIPK4 and HA-tagged ubiquitin that could only form K48-linked chains and then stimulated the cells with wnt3a. We observed that in wild-type cells RIPK4 was strongly modified by K48-linked ubiquitin reaching a maximum after one hour of wnt3a stimulation. In contrast, in the absence of A20, K48-linked ubiquitin on RIPK4 was decreased particularly at later time points (Fig 4C). Furthermore, reconstitution of A20 knockout RKO cells with wild-type A20 rescued K48-linked RIPK4 ubiquitination and induced RIPK4 degradation after wnt3a stimulation, while reconstitution with an A20 zinc-finger 4 mutant unable to support K48-linked ubiquitination did not (S6 Fig). Taken together, these data suggest that A20 normally supports RIPK4 K48-linked ubiquitination and degradation, serving as a negative regulator of wnt-signaling.

## Discussion

The ubiquitin editing enzyme A20 has multiple critical negative regulatory roles in the context of inflammation. Indeed, A20 has been linked to numerous inflammatory and autoimmune diseases [36] and cell type specific A20-deficiency in mice recapitulate many features of these diseases [37–42]. Regulation of inflammatory signaling by A20 often proceeds through post-translational modification of a family of proximal signaling kinases, the RIP kinase family. We previously showed that A20 has an unexpected role in regulating the wnt-stimulated beta-catenin pathway [26]. Furthermore, we noted that mice with an epithelial specific deletion in A20 under an APCmin background, develop a higher tumor burden than control APCmin mice. In this manuscript we explore the possible molecular mechanism behind this and provide further evidence of the potential physiologic consequence of this interaction.

First, we show that A20 deficiency in epithelial cells dysregulates wnt3a-dependent gene expression. One pathway that appears to be perturbed is the EGFR signaling pathway, one with critical importance in colorectal cancer development [43]. Further studies are needed to determine whether this aberrant gene expression contributes to the increased tumor burden seen in IEC-specific A20 knockout mice crossed to the APCmin background. Our studies may also indicate a potential interaction between the EGF and wnt-signaling pathways. Additionally, since EGF-signaling antagonists are an important clinical tool used in treatment of several types of cancers including colon cancer [44], further studies are needed to determine whether tumors with A20 mutations may be potential targets for EGFR inhibition.

Next, we provide evidence that A20 interacts with the fourth member of the RIP kinase family, RIPK4. Interestingly, RIPK4 has recently been shown to be an important positive regulator of the wnt signaling pathway. Given the known interactions between A20 and RIP kinases 1, -2, and -3, this may suggest that the interaction between A20 and the RIP kinase family is a conserved mechanism through which A20 exerts its regulatory functions. This also might suggest that A20 serves as a regulatory node through which multiple cell-intrinsic and cell-extrinsic signals might be integrated. Dysfunction or mutation of A20 might therefore lead to dysregulated responses to inflammation or homeostatic signaling.

A20 may exert its negative regulatory effects through both catalytic and non-catalytic pathways. Mechanism of A20 activity includes both ubiquitin modification and non-catalytic mechanisms [7–9,45]. Indeed, the deubiquitinase function of A20 is not required for NFkB signaling [46]. In this report, we demonstrate that similar to its effect on RIPK1 in the TNF-signaling pathway, A20 appears to support K48-linked ubiquitination of RIPK4. Further studies will be required to investigate whether other ubiquitin modifications of RIPK4 are important for its activity and function.

Overall, we further delineate a role for A20 in regulating a pro-carcinogenic pathway. Given its known role in regulating inflammation, this suggests an important dual function in regulating both inflammation and cancer development.

## Supporting information

S1 Fig A20 knockout cell line validation. A) qPCR of A20 transcript from wild-type (WT 293) and A20 knockout (KO 293) cell lines. B) qPCR of A20 transcript from wild-type (WT RKO) and A20 knockout (KO RKO) cell lines. C) Wild-type (WT 293) or knockout 293 cells (KO 293) were transfected with an NFkB-luciferase reporter and then stimulated with TNF for 8 hours. Luciferase activity was measured and normalized to Renilla luciferase. D) Wild-type (WT 293) or knockout 293 cells (KO 293) were transfected with a TCF4-luciferase reporter and then stimulated with wnt3a for 8 hours. Luciferase activity was measured and normalized to Renilla luciferase. E) Western blot showing protein expression of wild-type A20 constructs used in Fig 1E. ** = p < 0.05. Each panel is representative of at least three independent experiments.
(TIF)

S2 Fig Top ten differentially upregulated and downregulated transcripts in A20 siRNA knockdown RKO versus control siRNA knockdown RKO cells stimulated with wnt3a for 24 hours. (TIF)

S3 Fig Domain architecture comparison of RIPK1, RIPK2, RIPK3, and RIPK4. Sequence identity and similarity determined by protein-protein BLAST.
(TIF)

S4 Fig Expression of A20 or RIPK4 after stimulation or siRNA knockdown. A) RKO cells stimulated with TNF or wnt3a for the indicated timepoints were lysed and probed for A20, RIPK4, or GAPDH as a loading control. B) Efficiency of RIPK4 siRNA knockdown in RKO cells shown by western blot. GAPDH shown as a loading control. C) Western blot showing protein expression of RIPK4 K51R constructs used in Fig 4B. GAPDH shown as a loading control. Each panel is representative of at least three independent experiments.
(TIF)

S5 Fig A20 and RIPK4 truncation mutants. Full-length A20 (A20 FL), A20 N-terminal truncation mutant (A20 NT), A20 C-terminal truncation mutant (A20 CT). Ovarian-tumor like domain (OTU). Zinc fingers 1–7 (Z1-Z7). Full-length RIPK4 (RIPK4 FL), N-terminal RIPK4 mutant (RIPK4 NT), C-terminal RIPK4 mutant (RIPK4 CT). Kinase domain (KD), Intermediate domain (ID), Ankyrin repeat domain (ANK). Numbers denote amino acid number.
(TIF)

S6 Fig Reconstitution of A20 knockout RKO with wild-type or zinc-finger 4 mutant A20. A20 knockout RKO cells were transfected with control vector (CNTL), Flag-tagged wild-type A20 (WT A20), or a FLAG-tagged zinc-finger 4 mutant A20 (A20 ZF4), in addition to MYC-tagged RIPK4 and HA-tagged K48-only ubiquitin and then stimulated with wnt3a for 30 minutes. MYC-tagged RIPK4 was immunoprecipitated and then blotted for HA-tagged K48-only ubiquitin. Inputs shown below. GAPDH shown as a loading control. Representative of three independent experiments.
(TIF)
