# Supplementary figures and images for: A20 regulates canonical wnt-signaling through an interaction with RIPK4
Source: PLoS One. 2018 May 2;13(5):e0195893. doi: 10.1371/journal.pone.0195893 (PMC5931457; doi:10.1371/journal.pone.0195893)

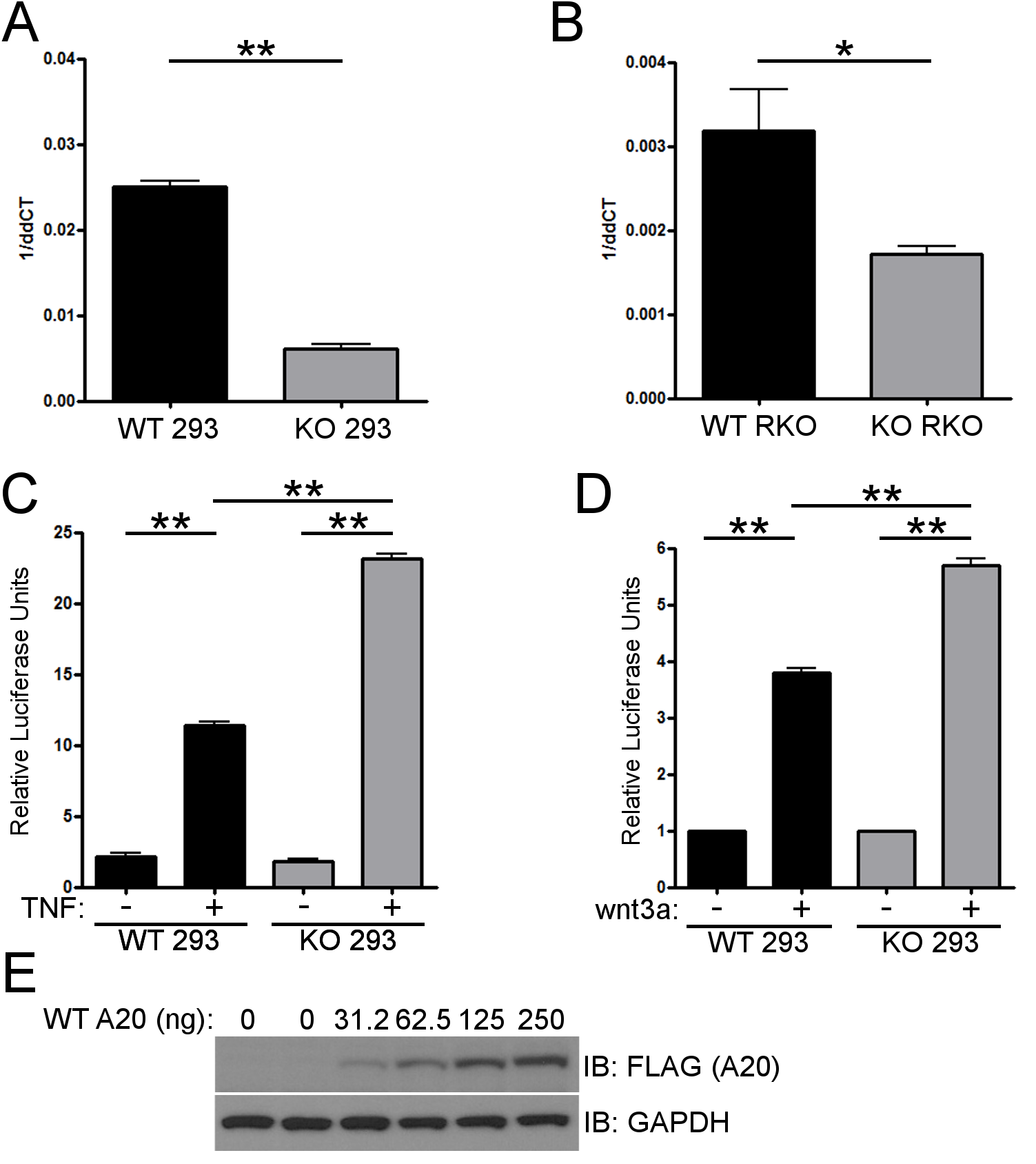

Supplement: S1 Fig — A) qPCR of A20 transcript from wild-type (WT 293) and A20 knockout (KO 293) cell lines. B) qPCR of A20 transcript from wild-type (WT RKO) and A20 knockout (KO RKO) cell lines. C) Wild-type (WT 293) or knockout 293 cells (KO 293) were transfected with an NFkB-luciferase reporter and then stimulated with TNF for 8 hours. Luciferase activity was measured and normalized to Renilla luciferase. D) Wild-type (WT 293) or knockout 293 cells (KO 293) were transfected with a TCF4-luciferase reporter and then stimulated with wnt3a for 8 hours. Luciferase activity was measured and normalized to Renilla luciferase. E) Western blot showing protein expression of wild-type A20 constructs used in Fig 1E. ** = p < 0.05. Each panel is representative of at least three independent experiments. (TIF) [file pone.0195893.s001.TIF]

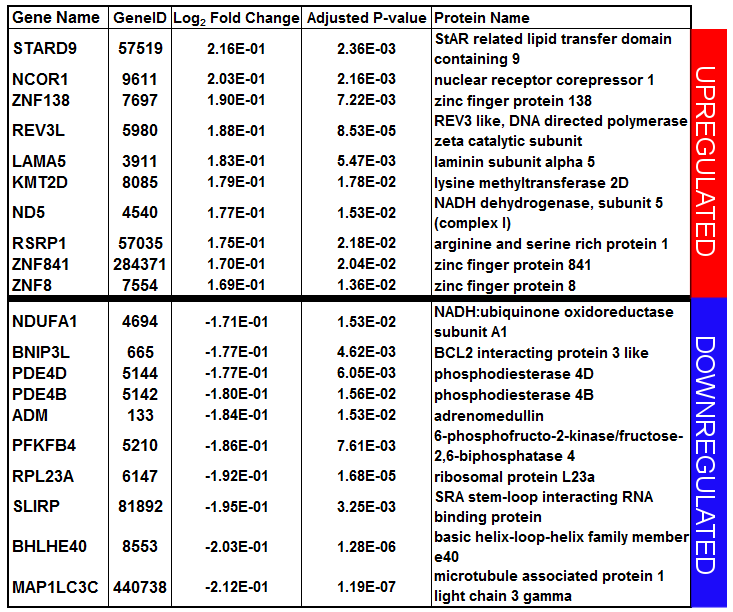

Supplement: S2 Fig — (TIF) [file pone.0195893.s002.tif]

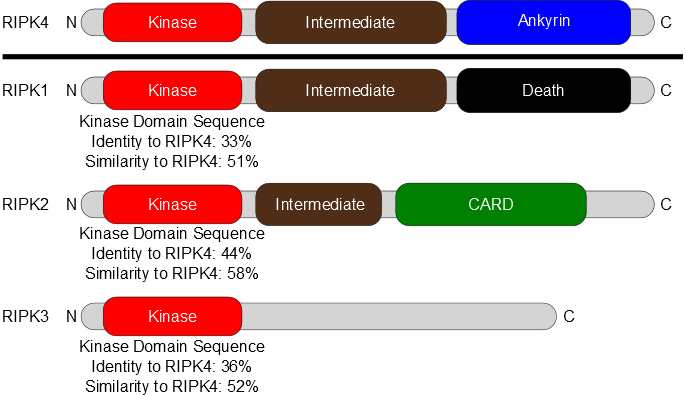

Supplement: S3 Fig — Sequence identity and similarity determined by protein-protein BLAST. (TIF) [file pone.0195893.s003.tif]

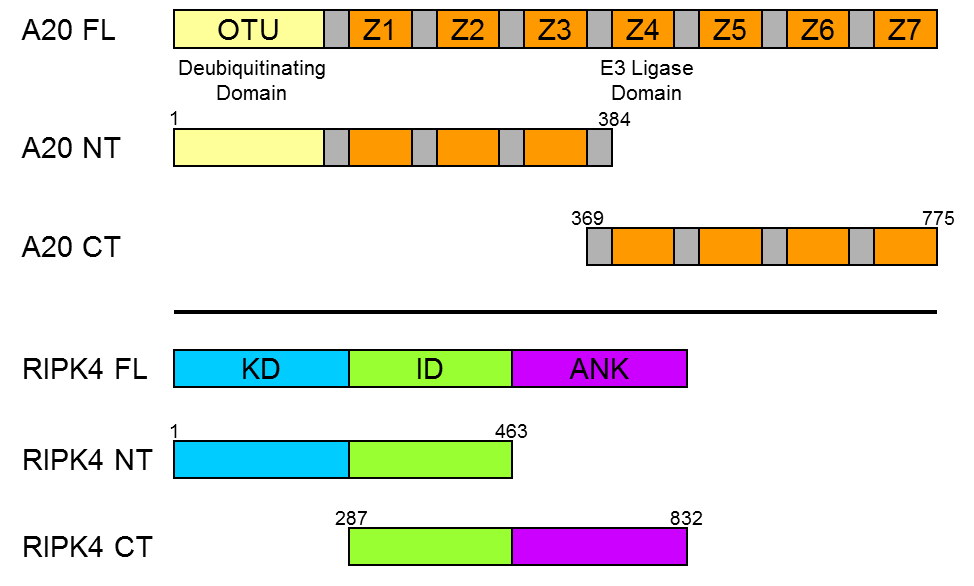

Supplement: S5 Fig — Full-length A20 (A20 FL), A20 N-terminal truncation mutant (A20 NT), A20 C-terminal truncation mutant (A20 CT). Ovarian-tumor like domain (OTU). Zinc fingers 1–7 (Z1-Z7). Full-length RIPK4 (RIPK4 FL), N-terminal RIPK4 mutant (RIPK4 NT), C-terminal RIPK4 mutant (RIPK4 CT). Kinase domain (KD), Intermediate domain (ID), Ankyrin repeat domain (ANK). Numbers denote amino acid number. (TIF) [file pone.0195893.s005.TIF]

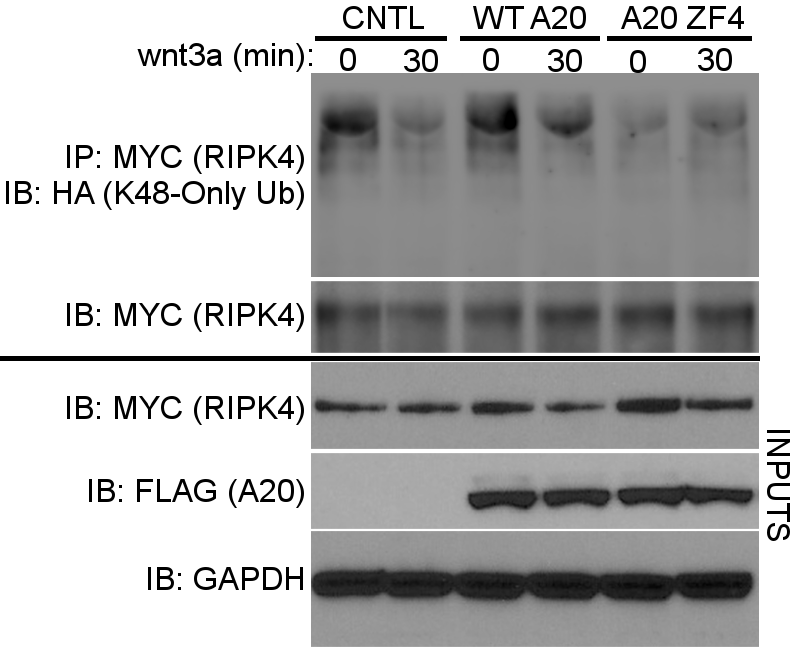

Supplement: S6 Fig — A20 knockout RKO cells were transfected with control vector (CNTL), Flag-tagged wild-type A20 (WT A20), or a FLAG-tagged zinc-finger 4 mutant A20 (A20 ZF4), in addition to MYC-tagged RIPK4 and HA-tagged K48-only ubiquitin and then stimulated with wnt3a for 30 minutes. MYC-tagged RIPK4 was immunoprecipitated and then blotted for HA-tagged K48-only ubiquitin. Inputs shown below. GAPDH shown as a loading control. Representative of three independent experiments. (TIF) [file pone.0195893.s006.TIF]
